# Comparative Toxicokinetics and Biomarker Responses of Typical Psychiatric Pharmaceuticals in *Daphnia magna*

**DOI:** 10.3390/toxics13060481

**Published:** 2025-06-06

**Authors:** Haohan Yang, Hao Xing, Zhuoyu Chen, Linghui Kong, Hanyu Jiang, Tengyi Zhu

**Affiliations:** College of Environmental Science and Engineering, Yangzhou University, Yangzhou 225127, China; mz120241359@stu.yzu.edu.cn (H.X.); mz120231301@stu.yzu.edu.cn (Z.C.); mz120231312@stu.yzu.edu.cn (L.K.); 231602108@stu.yzu.edu.cn (H.J.)

**Keywords:** psychiatric pharmaceuticals, uptake, depuration, toxicokinetics, biological effects

## Abstract

The widespread availability and pseudo-persistence of typical psychiatric pharmaceuticals (PDs) can have serious impacts on aquatic ecosystems and even human health. However, the toxicokinetics of typical PDs and the corresponding enzymatic biomarker responses are unclear. In this study, eight typical PDs [carbamazepine (CBZ), citalopram (CIT), sertraline (SER), venlafaxine (VLF), amitriptyline (AMT), chlorpromazine (CPM), quetiapine (QTP) and clozapine (CLZ)] were selected to study the uptake, depuration and biological effects of PDs in *Daphnia magna*. The results found that the uptake rates (*K*_u_) were in the sequence of VLF < QTP < CBZ < CLZ < CIT < AMT < SER < CPM, while the depuration rates (*K*_d_) were in the order of CLZ < AMT < CIT < SER < QTP < CBZ < CPM < VLF. Correspondingly, the bioconcentration factors (BCFs) followed on as VLF < QTP < CBZ < CIT < AMT < CLZ < SER < CPM. Both pH-dependent octanol–water partition coefficients (log *D*_ow_) and liposome–water partition coefficients (log *D*_lip-w_) exhibited positive correlations with the log BCF of PDs (*p* < 0.05), indicating the important roles of ionization degree and biological phospholipid contents on bioconcentration. Superoxide dismutase (SOD) activities were evidently induced in the SER and CPM groups, while ethoxyresorufin-O-deethylase (EROD) and glutathione-S-transferase (GST) activities were significantly induced only in the CBZ group. Acetylcholinesterase (AChE) activity was obviously induced by CBZ, SER and AMT, with levels 1.73, 1.62 and 2.44 times that of the control group (*p* < 0.05). The *K*_u_ of PDs, oxidative stress and metabolic level of *D. magna* combine to affect BCF levels together. In conclusion, this study contributes to a better understanding of the toxicokinetics and biochemical responses of PDs in *D. magna* and potential mechanisms of action, which may allow for a better assessment of their environmental health risks to aquatic ecosystems.

## 1. Introduction

In recent years, pharmaceutical compounds widely present in the environment have received international attention due to their pseudo-persistence [1,2]. Psychiatric drugs (PDs), such as antiepileptics, antidepressants and antipsychotics, are among the most prescribed active substances globally, which can directly act on the central nervous system and disrupt neuro-endocrine signaling [3,4]. The globe consumption of PDs has increased with the volume of sales at an average annual rate of 4.08% in the last two decades [5]. To date, PDs have frequently been detected in waste water, and it has been shown that PDs end up in environmental matrices, including surface and ground waters and sediments. Moreover, PDs are engineered to interact with biological targets, including receptors and enzymes, which are frequently evolutionarily conserved in non-target species. It is considered that the wide existence of PDs might cause serious impacts on ecosystems and even human health.

In fact, the bioaccumulation potential of typical PDs has been reported due to their ubiquitous presence in aquatic environments. For example, field investigations found that the bioaccumulation factors (BAFs) of the antidepressant sertraline (SER) and the antidepressant amitriptyline (AMT) in wild *Lasmigona costata* were 32,022 and 6028 L/kg, respectively [6]. The uptake and accumulation of the antiepileptic carbamazepine (CBZ) by *Eisenia fetida* causes secondary poisoning in predator birds [7]. A series of investigations found the selective uptake and high bioaccumulation of the antidepressant citalopram (CIT), the antidepressant venlafaxine (VLF) and CBZ in *Mytilus galloprovincialis*, *Morone saxatilis* and Hirudinea, respectively [8,9,10]. However, toxicokinetics (TK) processes primarily consist of uptake, depuration and biotransformation, which describe the temporal dynamics of pollutant concentrations within organisms [11,12,13]. Therefore, conducting TK studies could facilitate the comprehension of the fate of PDs in organisms and their physiological processes.

Meanwhile, the toxicological effects induced by PDs have constantly been raised, including in the nervous, antioxidant and metabolic systems. For instance, AMT could induce acetylcholinesterase (AChE) activity in *Danio rerio* (*D. rerio*) and decrease glutathione-S-transferase (GST) activity [14]. The significant inhibition of superoxide dismutase (SOD) activity and relative *sod1* mRNA expression induced by the antipsychotic clozapine (CLZ) and SER were observed in zebrafish larvae after exposure for 72 h [15,16]. Likewise, the antipsychotic quetiapine (QTP) [17] and the antipsychotic chlorpromazine (CPM) [18] induced dose-dependently attenuated neuronal injury and imbalance of oxidative stress, respectively, in *Mytilus galloprovincialis*. To date, some studies have reported that the bioconcentration factor (BCF) does not correlate with the octanol–water partition coefficient (*K*_ow_) of ionizable organic compounds, but instead with pH-dependent octanol–water distribution ratio (*D*_ow_) and liposome–water distribution ratio (*D*_lip-w_) [19]. However, to the best of our knowledge, systematic studies on the bioconcentration and biological effects of different types of PDs are still limited.

As a typical aquatic invertebrate, *Daphnia magna* (*D. magna*) plays an important role in the energy flow and material cycle of aquatic ecosystems, and has been widely used in toxicological research for environmental risk assessment. In this study, the uptake, depuration and biomarker responses of eight typical PDs were studied using *D. magna* as a test animal. The relationship between chemical biomarkers [SOD, GST, ethoxyresorufin-O-deethylase (EROD), AChE] and bioconcentration dynamics parameters [the uptake rate (*K*_u_), the depuration rate (*K*_d_), BCF] was further analyzed. To a certain extent, this study has filled in the toxicokinetics and biomarker responses of PDs in aquatic organisms. This study will help explore the ecological risk of PDs and provide theoretical support for environmental management.

## 2. Materials and Methods

### 2.1. Chemicals and Reagents

Eight different kinds of PDs were purchased from J&K Scientific Ltd. (Beijing, China). The specific physicochemical properties and pharmacokinetic parameters are presented in Table 1. Correspondingly, the internal standards amitriptyline-d_3_, carbamazepine-d_10_ and clozapine-d_4_ were supplied by Dr. Ehrenstorfer (Augsburg, Germany). All the organic solvents were high-performance liquid chromatography (HPLC)-grade. Chemical stock solutions were prepared in methanol and stored at −20 °C. Deionized water was obtained using a Milli-Q purification system (Millipore, Milford, MA, USA).

### 2.2. Experimental Animals and Acclimation

The test animal *D. magna* and green algae *Scenedesmus obliquus* (FACHB-13) were obtained from the Institute of Hydrobiology, Chinese Academy of Science, and have been cultivated in our laboratory for at least five years [20,21,22]. The animals were cultured in Elendt M4 medium according to OECD 211 (Organization for Economic Cooperation and Development, 2012) [23]. The standard culture was renewed twice a week and maintained at 25 ± 0.5 °C with a natural light/dark cycle. The feed for *D. magna* cultivation, *Scenedesmus obliquus*, was given thrice a week with a density of 0.1–0.2 mgC/animal/day. Seven-day-old daphnids were used for exposure in this study.

### 2.3. Aqueous Uptake and Depuration of PDs

The experiments for bioaccumulation kinetics included the two stages of uptake and depuration. A total of one control and eight exposure groups were set up. For the uptake stage, considering the different toxicity of the targeted compounds in bioaccumulation, the exposure concentration of each compound was selected as its 10% 48 h LC_50_ for daphnids, as shown in Table 2. The LC_50_ values were obtained from related reports or the ECOSAR prediction. Then, 200 selected adult individuals (seven days old) were added to 500 mL of the exposure solution, and the pH of the solution was adjusted to 7.5 by adding HCl or NaOH as required. The co-solvent methanol concentration was <0.1 mL/L. The exposure concentrations of the individual compounds were measured at predetermined sampling intervals during the duration of the uptake (Table S2). The sampling interval was determined based on the results of per-experiment. At each sampling time point, thirty daphnids were randomly collected and rinsed using deionized water three times, dried with glass microfiber filters (GF/F; Whatman, Maidstone, UK) and then transferred to a 1.5 mL centrifuge tube to obtain their wet weight (ww). Meanwhile, the water samples were filtered through a 0.22 µm membrane filter and stored in 1.5 mL glass vials for subsequent analysis. If the exposure time of any chemical exceeded 24 h, the exposure solution was renewed. All the samples were processed in triplicate. For the depuration period, at the end of the uptake phase, the exposed daphnids in the remaining beakers were transferred to clean Elendt M4 medium. Then, the animal and water samples were collected depending on the time of sampling. No feeding was provided throughout the experiments, with the exception of the treatments involving citalopram and sertraline, for which *Scenedesmus obliquus* was supplied at 0.1 mgC/animal/day. Meanwhile, fifty animals were collected randomly at the end of uptake and depuration duration for biomarker analysis. All the water and daphnid samples were stored at 4 and −80 °C.

### 2.4. Sample Pretreatment and Quantification of Target PDs

According to the methods of Yang et al. [24] and Nkoom et al. [25], ultrasonic extraction in combination with a Waters Acquity ultra-performance liquid chromatography tandem mass spectrometry system (UPLC-MS/MS; Waters, Milford, MA, USA) was used for PD determination. An ACQUITY UPLC BEH-C18 column (100 mm × 1.7 μm × 2.1 mm; Waters, USA), positive electrometer ionization (ESI^+^) source and multiple reaction monitoring (MRM) were employed for the separation, identification and quantification of the compounds, respectively. The specific information of the extraction and instrumental analysis are described in Text S1 and Table S1, respectively.

### 2.5. Biomarker Analysis

Pre-frozen daphnid samples were homogenized in 0.1M potassium dihydrogen phosphate buffer (pH 7.4) and subsequently centrifuged at 4 °C for 15 min (15,000× *g*). The supernatant was separated, and we determined the AChE, SOD, GST and EROD activities using commercial kits (Nanjing Sunshine Biotechnology Co., Ltd., Nanjing, China). The analysis of AChE activity was based on the approach of Ellman et al. [26] at 405 nm. The qualification of SOD activity referred to the method of Marklund and Marklund [27] at 420 nm. The GST activity was determined based on the method of Habig and Jakoby [28] at 340 nm. EROD activity determination was based on the amount of reaction product of resorufin at excitation and emission wavelengths of 530 and 585 nm, respectively [29]. Protein content was measured according to the method of Bradford [30] using serum albumin as a standard. The enzyme activities in all the samples were analyzed in triplicate for each pool using a microplate reader Multiskan FC 100 (ThermoFisher Scientific, Waltham, MA, USA). All the active units of the enzymes were expressed as nmol/mg total protein/min.

### 2.6. Quality Assurance and Quality Control

The quantification of the target compounds was based on the isotope dilution method. The limits of detection (LODs) and the limits of quantitation (LOQs) of the target PDs were 0.09–0.67 and 0.27–2.01 ng/g ww, respectively. The recovery rates of each compound varied from 65.1 to 166.7% and 58.7 to 89.5% at spiked concentrations of 10 and 100 ng/g ww, respectively. The detailed analysis and results of the matrix recoveries, LODs and LOQs for the eight PDs are provided in Text S2 and Table S2.

### 2.7. Bioaccumulation Dynamic Model

The uptake and depuration process of the targeted compounds by daphnids was simulated by the one-compartment toxicological kinetic mode Formula (1)

(1) $$\frac{dC_{\mathrm{organism}}}{dt}=k_{u}\cdot C_{\mathrm{water}}(t)-k_{d}\cdot C_{\mathrm{organism}}(t)$$

where *t* is the exposure time (h) and *C*_organism_ is the body burden of the targeted compound in *D. magna* (μg/kg). The body burden is the concentration of targeted PDs in *D. magna* during exposure. *C*_water_ is the concentration of the targeted compound in water (μg/L), and *k*_u_ and *k*_d_ are the uptake rate constant (L kg^−1^ h^−1^) and depuration rate constant (h^−1^) of the targeted compound, respectively. At the beginning of the experiment, the body burden was 0; that is, *C*_organism_ (0) = 0. Assuming that the concentration of the target compound in the water remains constant, the concentration of the substance in the bodies of the *D. magna* can be expressed as

(2) $$C_{\mathrm{organism}}(t)=\frac{k_{u}}{k_{d}}\cdot C_{\mathrm{water}}(1-e^{-k_{d}t})$$

By substituting the data of the recovery stage into the first-order metabolism model, the elimination rate constant *k*_d_ can be expressed as:

(3) $$C_{\mathrm{organism}}(t)=C_{i\;}e^{-k_{d}\;t}$$

where *C*_i_ represents the body burden of the targeted compound at the beginning of the fresh water recovery stage (μg/kg).

Based on toxicological kinetic data or equilibrium concentration, the bioconcentration factor (BCF, L/kg) could be calculated using Formula (4):

(4) $$\mathrm{BCF}=\frac{k_{u}}{k_{d}}=\frac{C_{\mathrm{organism}}}{C_{\mathrm{water}}}$$

### 2.8. Statistical Analysis

One-way analysis of variance was used for statistical analysis, and Duncan’s post hoc test was used for significant difference analysis among different groups, with *p* < 0.05 showing significant differences. All data analysis was processed using OriginPro 8 (OriginLab Corporation, Northampton, MA, USA) and SPSS (ver. 17.0, SPSS Company, Chicago, IL, USA).

## 3. Results and Discussion

### 3.1. Uptake and Depuration Kinetics of PDs in D. magna

The acute toxicities of the eight PDs were obtained at pH 7.5 (Table 2). CPM and SER elicited high toxicity, with LC_50_ values of 0.73 and 0.98 mg/L, respectively. QTP, CLZ and AMT exhibited moderate toxicity, with LC_50_ values of 1.80, 2.91 and 6.20 mg/L, respectively. CIT, VLF and CBZ elicited low toxicity, with LC_50_ values of 13.72, 100.3 and 149 mg/L. Similarly, the LC_50_ results for CPM and SER were compared to previous studies which showed LC_50_ values of 1.81 mg/L [31] and 0.126 mg/L [32] in *D. magna*, respectively. The acute toxicity levels were also comparable to the results of Minguez et al. [33], who found EC_50_ values to progress from most to least in the order of SER > AMT > CIT > VLF. In fact, these PDs belong to the family of Cationic Amphiphilic Drugs (CADs). These CADs exhibit targeted interactions with cell membrane components [34,35]. The mechanism involves protonated groups on the CADs catalyzing the acid hydrolysis of ester functional groups to produce fatty acids and single-chain lipids that induce membrane destabilization. Changes in the toxicity of antidepressants at the lysosomal membrane may depend on the ability of their derivatives to catalyze the acidic hydrolysis of the ester group, a process determined by the presence or absence of optimal cation–π interactions. Considering the accurate quantification of PDs’ body burdens and the absence of adverse impacts in the uptake experiments, the exposure concentrations chosen were comparable to, or up to approximately ten times higher than, the environmentally relevant concentrations [4,36]. During exposure, the concentrations of the eight targeted PDs in water decreased by less than 20% (17.48–19.44%) compared to the nominal concentration, indicating that the substances were relatively stable.

**Figure 1:**
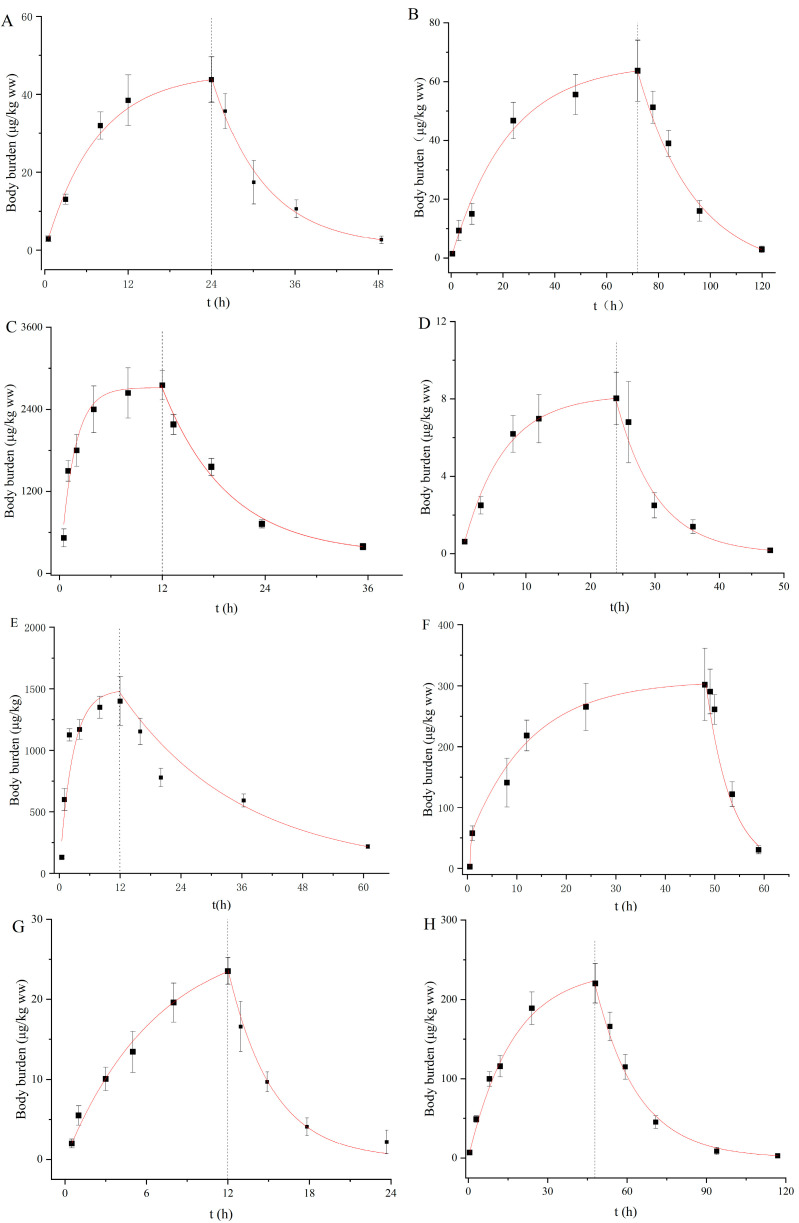
Body burden of targeted pharmaceuticals in *D. magna* during uptake and depuration periods. ((**A**): CBZ; (**B**): CIT; (**C**): SER; (**D**): VLF; (**E**): AMT; (**F**): CPM; (**G**): QTP; (**H**): CLZ).

The uptake and depuration kinetics of the eight PDs in *D. magna* are described in Figure 1, and the rate constant and BCF were calculated by the one-compartment first-order toxicological model (Table 2). The body burdens of the PDs increased rapidly to an equilibrium state within exposure durations of 120 h for all the compounds. Similarly, such rapid steady increases were observed for roxithromycin, propranolol and diclofenac in *D. magna* in our previous study [37,38]. With regard to the recovery stage, most of the PDs were rapidly removed from the daphnid bodies and then slowly decreased (Figure 1). The coefficients of determination (*r^2^*) were between 0.88 and 0.95. The *K*_u_ values ranged from 1.53 to 180.00 L/kg/h in a descending order of VLF < QTP < CBZ < CLZ < CIT < AMT < SER < CPM (Table 2). In fact, the *K*_u_ values of these PDs were not correlated with the log *K*_ow_ values, but instead depended on the log *D*_ow_ values at environment-related pH and the degree of ionization. Although SER, AMT and CPM exist primarily as ions (>98%) at pH 7.5, these compounds have higher log *D*_ow_ values of 3.62, 3.02 and 3.60, respectively. Hence, they were rapidly absorbed by *D. magna*, with *K*_u_ values of 48, 152 and 360 L/kg/h. Conversely, CLZ, QTP and CBZ take mainly neutral forms at pH 7.5 and have lower log *D*_ow_ values, exhibiting slower *K*_u_ values of 6.85, 3.49 and 6.78 L/kg/h, respectively. As the main route of uptake for exogenous chemicals, passive diffusion has been found to be driven by chemical lipophilicity, which is related to both neutral species and ionic forms [9,19]. Niu et al. [39] also indicated the negligible role of charged species of PDs in their partitioning behaviors. In our study, *K*_u_ demonstrated a generally comparable trend to log *D*_ow_, but this observation did not occur for all compounds. Alternatively, structural proteins, phospholipids and serum albumin can be used as potential additional biosorption phases to adsorb ionized chemical species [40]. Furthermore, active transport may occur as a specific membrane carrier. Netherton et al. [41] found that SSRIs could be selectively bioaccumulated by fish, in whom active transport mechanisms may contribute to the uptake of these pharmaceuticals. Thus, the uptake mechanism of PDs by aquatic organisms is still unclear to a large extent, with more research needed to elucidate this.

The *K*_d_ values were in the range of 0.01 to 0.25 h^−1^ and in the descending order of CLZ < AMT < CIT < SER < QTP < CBZ < CPM < VLF (Table 2). CPM, VLF and QTP were relatively rapidly eliminated due to their high *K*_d_ values, and their body residues reduced by more than 80% within 12 h during the recovery phase in clean water (Figure 1). Similarly, the *K*_d_ values for SER and AMT in our study were within the same order as the observations of Ivankovic et al. [42] and Zhang et al. [19] who showed values of 0.078 and 0.042 h^−1^ in zebrafish, respectively. Chen et al. [43] found that the biotransformation rate constant was an important parameter affecting the bioaccumulation potential of chemicals. It has been found that the rapid transformation of VLF to its three main metabolites (O-desmethylvenlafaxine, N-desmethylvenlafaxine and NO-didesmethylvenlafaxine) in bivalves under the action of cytochrome P4502D6 on N-desmethyl metabolites occurs immediately [44]. Regarding CBZ metabolization, Boillot et al. [45] detected acridine and carbamazepine-10, 11-epoxide metabolites when bivalves were exposed to 100 µg/L carbamazepine in water. However, metabolites of AMT were detected in mammal animals [46] and gilt-head bream [47], but not in *D. magna.*

### 3.2. Bioconcentration Factors

As a result, the BCF values calculated by toxicokinetic-state and steady-state concentrations were between 23.58–1084.34 L/kg ww and 9.45–1576.27 L/kg ww, respectively (Table 2). The comparable results of the two algorithms indicated the effectiveness of the experiments. As a result, the BCF values of the eight PDs were ordered as follows: VLF < QTP < CBZ < CIT < AMT < CLZ < SER < CPM. According to the EUHA guidance, a chemical can be defined as bioaccumulative or very bioaccumulative when its BCF value is over 2000 or 5000, respectively. The BCFs in this study of less than 2000 illustrated that the bioconcentration potentials of the selected PDs were relatively low in *D. magna*.

Furthermore, a correlation was established between BCFs and the log *K*_ow_ values of the substances, taking into account the importance of log *K*_ow_ as an indicator of the bioaccumulation of lipophilic organic chemicals [48]. As the results show (Figure S1), there was no statistically significant correlation for log *K*_ow_ values of 1.94–5.41 (*p* > 0.05, *r*^2^ = 0.4058). Comparably, previous studies have also found that the bioconcentration of PDs is poorly correlated with their lipophilicity [3,49]. Similarly to other ionized pharmaceuticals, the pH of the exposure solution is a vital factor for PDs, and different pHs can affect the ionization degrees of the chemicals [50]. Hence, to better predict the BCF of the targeted PDs, we replaced *K*_ow_ with the pH-dependent *D*_ow_ to predict BCF. Significant correlations were observed between the log BCF values and the log *D*_ow_ values (log BCF = 0.658, log *D*_ow_ + 0.578, *p* < 0.05, *r*^2^ = 0.6004). The log *D*_ow_ values of the eight targeted PDs decreased to 0.61-3.62 at pH 7.5, confirming the regulation effects of ionization degree on the lipophilic adsorption of the target compounds in *D. magna*.

**Figure 2:**
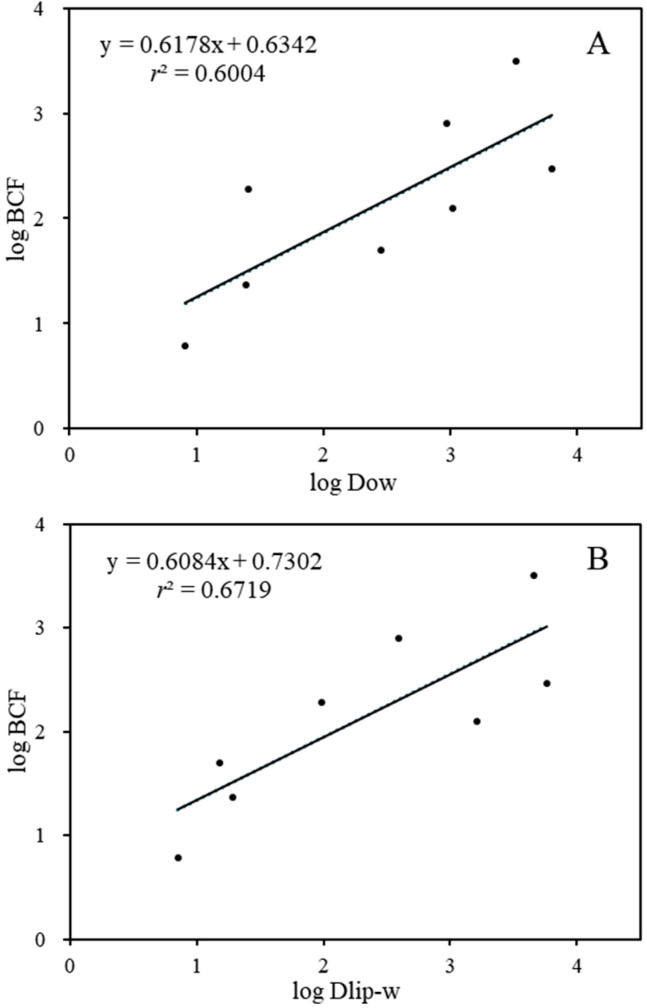
The relationship between log BCF and log D_ow_ (**A**)/log D_lip-w_ (**B**).

While *D*_ow_ is primarily considered as indication the storage of lipids in an organism, recent studies have indicated that phospholipids play a crucial role in the distribution of ionized pharmaceuticals [51,52]. The movement of ions and polar molecules across biological membranes is primarily regulated by specialized proteins, including membrane-bound channels and transporters [53]. Thus, the liposome–water partition coefficient (*D*_lip-w_) was calculated and linearly fitted with BCF (log BCF = 0.54 log *D*_lip-w_ + 1.00). As shown in Figure 2B, the BCF values of the eight target compounds were significantly correlated with their *D*_lip-w_ values (*p* ˂ 0.05, *r*^2^ = 0.6719). Similarly, other studies have also revealed that the capacity for the bioconcentration of cations and anions is significantly greater than that predicted by octanol–water partitioning estimates [54,55]. Both cations and anions show a pronounced affinity for the phosphatidylcholine bilayer, a property primarily stemming from the hydrophobicity of the ions. Neutral (nonionizable) organic compounds can adsorb significantly to phospholipids and storage lipids, whereas ionized compounds adsorb predominantly to phospholipids [56,57]. In our study, all targeted the PDs were ionized chemicals except for CBZ, and the ionization degree of the positively ionized PDs was greater than 95%. Hence, the pH-dependent *D*_lip-w_ might be more appropriate than the corresponding *D*_ow_ for the description of the uptake of hydrophobically ionized compounds into biological membranes. In addition, the observations of the selective bioaccumulation of PDs in aquatic environments might suggest that carrier-mediated processes might affect the uptake routes [9,58]. The biological effects induced by PDs could also affect their bioconcentration potential. For instance, *D. magna* exposed to CBZ could reduce the levels of lysophospholipids and increase the levels of some glycerophospholipids and triacylglycerol species [59]. The SER- and VLF-exposed *D. magna* could show increased L-Valine and L-Glutamic acid neurotransmitter concentrations [60].

### 3.3. Biomarker Responses

**Figure 3:**
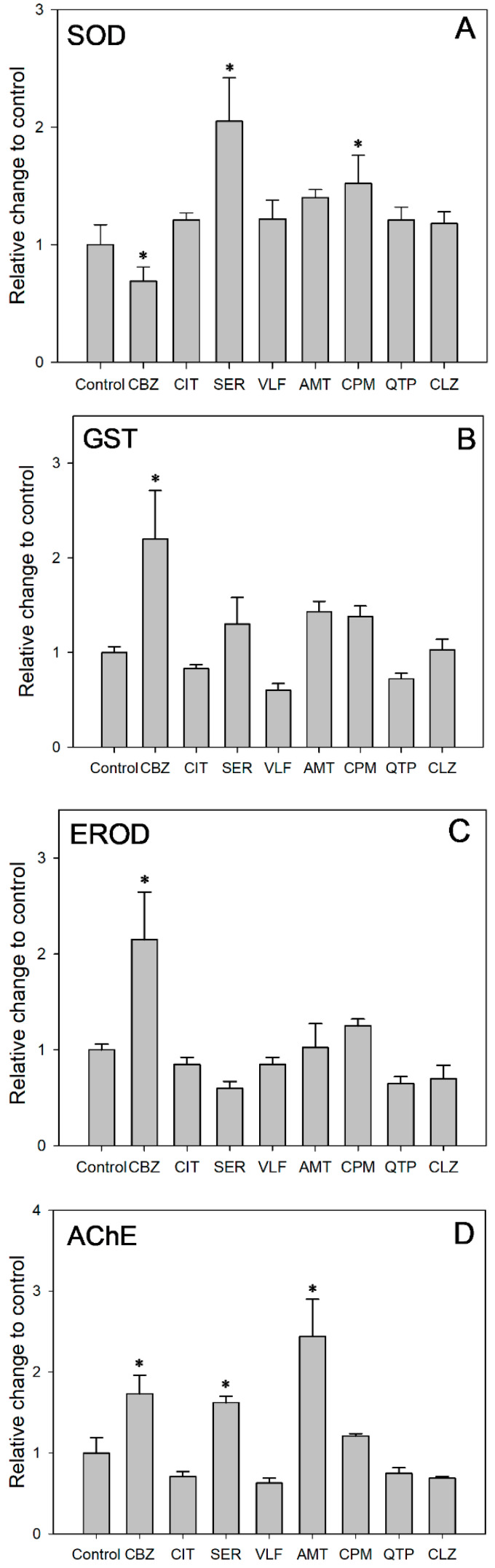
Effects of targeted PDs on activities of (**A**) SOD, (**B**) GST, (**C**) EROD and (**D**) AChE in *D. magna*. Each bar represent means ± SE. *: Significant difference between control group and treatment groups (*p* < 0.05).

The corresponding SOD activity was acquired after 48 h of exposure at the concentration of same acute toxicity (Figure 3A). SOD activity was induced in all the exposure groups except the CBZ group as compared to the control group. After 48 h of exposure at the concentration of same acute toxicity, SER and CPM induced SOD activities 2.05 and 1.52 times the value in the control, while CBZ inhibited SOD activities to 69% of the control value (ANOVA: F = 9.453; df = 8, 9; *p* = 0.001). These results suggested that increased SOD activity alleviated the oxidative stress induced by the targeted PDs. Antioxidant defenses were activated to eliminate the increased generation of reactive oxygen species (ROS) in cells. Moreover, the significantly increased SOD activity for the CPM and SER groups might be related to the higher *K*_u_ values of 180.0 and 48.0 L/kg/h, respectively. Higher uptake rates might exacerbate the accumulation of ROS and result in relatively severe oxidative stress. Comparatively, the suppressed SOD activity indicated that the test daphnids suffered from oxidative stress. There might be an accumulation of O^2−^, but not enough to induce SOD under the relatively slow *K*_u_ of 3.7 L/kg/h. Despite being exposed to the same concentration of acute toxicity, the different SOD activity responses indicated that the oxidative stress and damage of the PDs was *K*_u_-dependent. The uptake rate of pollutants should be considered in ecological risk assessment alongside exposure concentration.

As shown in Figure 3B, CBZ, SER, AMT and CPM induced GST activity, whereas CIT, VLF and QTP inhibited GST activity. Among them, the GST activity in the CBZ group increased remarkably, exceeding that of the control group by 2.20 times (ANOVA: F = 8.319; df = 8, 9; *p* = 0.002). Similarly, increased GST activity has been reported in *D. magna* exposed to CBZ [61], CIT [62], in zebrafish exposed to CBZ [63] and in *Carassius auratus* exposed to SER [64]. The GST enzyme family, which can bind reduced glutathione to exogenous or endogenous metabolites, is a crucial phase II detoxification enzyme [65]. As a critical component within biological organisms, the enzyme GST performs a pivotal function in diminishing the adverse effects of oxidative stress. This is achieved by the elimination of excessive ROS generation and the inhibition of lipid peroxidation [66]. The increased GST activity observed in *D. magna* in our study indicated that the phase II detoxification enzyme was involved in the metabolism of these compounds. Concurrently, the GST enzyme could counteract oxidative stress by scavenging ROS and inhibiting lipid peroxidation. In addition, the significant induction of GST in the CBZ group indicated that biotransformation might be an important process in *D. magna* at the maximum exposure concentration of 1.5 mg/L.

As shown in Figure 3C, CBZ, AMT and CPM induced EROD activity, whereas CIT, SER, VLF, QTP and CLZ slightly inhibited EROD activity. Among them, the EROD activity in the CBZ group increased remarkably, reaching a level 2.15 times higher than that observed in the control group (ANOVA: F = 11.529; df = 8, 9; *p* = 0.001). Likewise, an increase in EROD activity has been reported in *Cyprinus carpio* exposed to CBZ [67]. EROD, a phase I biotransformation enzyme, belongs to the CYP1A family of P450-dependent mono-oxygenase, which is regulated via the aryl hydrocarbon receptor (AhR) [68]. EROD enzyme activity can characterize CYP1A1 enzyme activity [69]. Thus, EROD is widely used as a biomarker to assess the toxic burden of environmental contamination [70]. Cytochrome P450 enzymes play a crucial role in metabolizing both exogenous and endogenous compounds [71]. The induction of phase I biotransformation enzymes by drugs, along with the associated oxidative stress, is a well-recognized phenomenon during drug metabolism [72]. In our study, the elevated EROD activity in *D. magna* demonstrated the involvement of phase I detoxification enzymes in metabolizing PDs. CYP1A1 may alleviate oxidative-stress-induced damage by scavenging excess ROS and suppressing lipid peroxidation. In addition, the significant induction of EROD in the CBZ group suggests that biotransformation may be an important pathway for *D. magna* at a maximum exposure concentration of 1.5 mg/L. The result is similar to that of the phase II biotransformation enzyme GST in this study.

AChE, a vital enzyme for nerve impulse transmission, catalyzes the hydrolysis of acetylcholine (ACh) into choline and acetic acid within the synaptic gaps of cholinergic synapses and neuromuscular junctions. In the present study, CBZ, SER and AMT significantly induced AChE activities to 1.73, 1.62 and 2.44 times the value in the control group (ANOVA: F = 23.570; df = 8, 9; *p* = 0.0001, Figure 3D). Likewise, an increase in AChE activity has been reported in crucian carp (*Carassius carassius*) exposed to CBZ [73] and in yellow catfish (*Tachysurus fulvidraco*) exposed to SER [74]. In fact, the ongoing apoptotic process in organisms [75,76] as well as the generation of free radicals and oxidative stress [77] have been linked to an increase in AChE activity. Increased AChE activity leads to the rapid degradation of the neurotransmitter acetylcholine, resulting in the downregulation of acetylcholine receptors and the subsequent impairment of cognitive functions in organisms [78]. Furthermore, the activity of AChE, which is suppressed by neurotoxic agents, serves as a common biomarker for monitoring environmental pollution [64,79,80]. A significant decrease (≥20%) in AChE activity is recognized as a clear indicator a toxicological impact of xenobiotic exposure, leading to reduced feeding activity and impaired swimming behavior in affected organisms. In our study, although AChE activity did not significant change compared to the control, AChE activity in the groups of CIT, VLF, QTP and CLZ was inhibited by 29%, 37%, 25% and 31%, respectively. These results indicated that the feeding activity and swimming rate of animals might be affected.

### 3.4. Correlation Analysis Between Toxicokinetic Parameters and Biochemical Response

**Figure 4:**
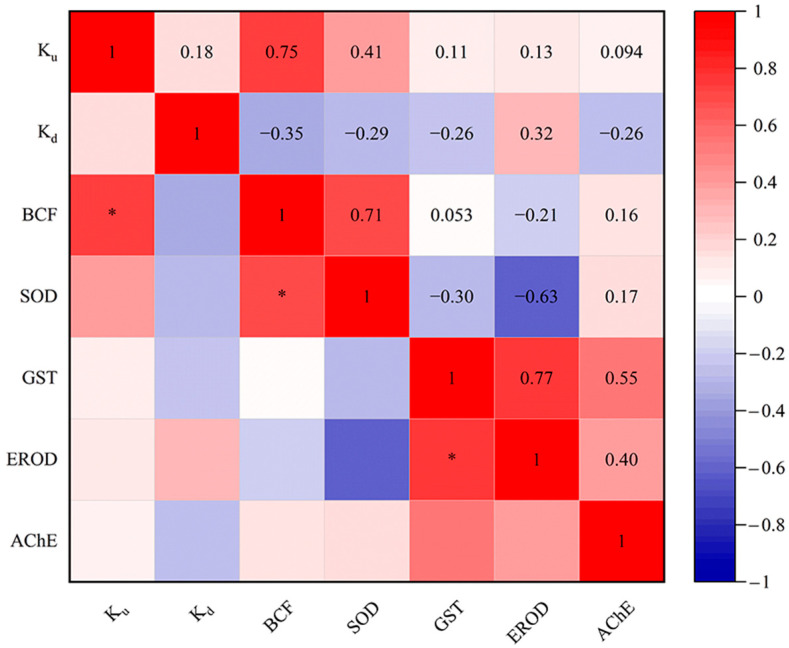
Relationships of *K*_u_, *K*_d_ and BCF values of targeted PDs for SOD, GST, EROD and AChE activities in *D. magna*. * *p* < 0.05.

In our study, systematical analysis between the toxicokinetic constants (*K*_u_, *K*_d_ and BCF) and biomarker responses (SOD, GST, EROD and AChE activities) of eight targeted PDs in *D. magna* were conducted (Figure 4). The results indicated a significant positive correlation between *K*_u_ and BCF values. By definition, *K*_u_ and BCF are directly proportional [48]. Likewise, a positive correlation between *K*_u_ and BCF toxicokinetic parameters has been reported in *D. magna* exposed to the dissolved uranium [81]. Additionally, the positive correlation between *K*_u_ and *K*_d_ in this study is similar to the results of Scheibener et al. [81]. Meanwhile, BCF was significantly positively correlated with SOD in our study. Elizalde-Velázquez et al. [82] found that BCF was positively correlated with the overproduction of ROS in *Danio rerio* adults exposed to guanylurea. The balance of ROS in vivo is disrupted in organisms exposed to pollutants [83,84]. In this case, the organism will produce antioxidants to balance the concentration of toxic ROS within its cells, such as the antioxidant enzymes SOD and GST [85]. Additionally, a significant positive correlation was identified between the activities of GST and EROD. In fact, CYP1A (EROD) and GST had a synergistic effect in the biotransformation process [86]. Changes in SOD, GST, EROD and AChE activities have been reported in crucian carp (*Carassius auratus*) exposed to ketoconazole [87]. However, few studies have been conducted to perform correlation analysis between toxicokinetic parameters and multilevel biomarkers using *D. magna* as a biological model. In the future, research in this area could be strengthened to better understand the effects of more PDs on aquatic organisms.

## 4. Conclusions

Taken together, this study systematically investigated the toxicokinetics and biomarker responses of eight typical PDs in *D. magna* to elucidate their underlying mechanisms. This study was conducted at an environmentally relevant pH of 7.5. All the targeted PDs (excluding CBZ) were ionizable, with positively ionized PDs exhibiting ionization degrees exceeding 95%. Acute toxicity showed that the PDs had specific interactions with cell membranes. The *K*_u_ values ranged from 1.53 to 180.00 L/kg/h. In fact, the *K*_u_ of these PDs is not related to the log *K*_ow_ value, but rather to the log *D*_ow_ value at the environmentally relevant pH and degree of ionization. Further research is needed to clarify PDs’ absorption mechanisms in aquatic organisms. *K*_d_ indicated distinct biological conversion efficiencies among PDs in *D. magna*. In this study, the relationships between log BCF and log *K*_ow_, log *D*_ow_, and log *D*_lip-w_ were determined, respectively. Factors that affect the bioaccumulation of ionizable organic compounds include the pH of the exposure solution and the phospholipids of *D. magna*. In addition, carrier-mediated processes and PD-induced biological effects are also potential influences. SOD activity varied with *K*_u_, suggesting the presence of *K*_u_-dependent oxidative stress. In ecological risk assessment, in addition to the exposure concentration, the absorption rate of pollutants should also be considered. The increase in EROD and GST activity in *D. magna* indicated that phase I and II detoxification enzymes, respectively, were involved in PD metabolism to reduce damage caused by oxidative stress. Especially in the CBZ-exposed group (1.5 mg/L), biotransformation may be an important pathway in *D. magna*. The change in AChE activity may affect the feeding activity and swimming speed of aquatic organisms. *K*_u_ was significantly and positively correlated with BCF, BCF with SOD, and GST with EROD. However, few studies have used *D. magna* as a biological model to perform a correlation analysis between toxicokinetics parameters and multilevel biomarkers. These findings may facilitate further understanding of the toxicokinetics and biomarker responses of PDs in aquatic organisms and their underlying mechanisms of action, which are important for environmental health risk assessment.

## Figures and Tables

**Table 1:** Physical and chemical properties of eight targeted PDs.

Type	Substance	Abb	CAS	Molecular Mass (g/mol)	Log *K*_ow_	p*K*a	Log *D*_ow_ (pH = 7.5)	Ionic Form	Proportion of Neutral Molecules (%)	Log *K*_lip-w_	Log *D*_lip-w_
Antiepileptics	Carbamazepine	CBZ	298-46-4	236.27	2.45	13.9	2.45	Neutral	100	1.18	1.18
SSRIs	Citalopram	CIT	59729-33-8	324.39	3.74	9.78	1.46	Positive	0.5	3.06	0.78
	Sertraline	SER	79617-96-2	306.2	5.29	9.16	3.62	Positive	0.8	5.32	3.66
SNRI	Venlafaxine	VLF	93413-69-5	277.4	3.2	10.1	0.61	Positive	3.7	2.28	0.85
TCA	Amitriptyline	AMT	549-18-8	313.86	4.92	9.4	3.02	Positive	0.5	4.77	3.21
	Chlorpromazine	CPM	50-53-3	318.86	5.41	9.3	3.60	Positive	2.0	5.47	3.76
Atypical antipsychotics	Quetiapine	QTP	111974-69-7	883.09	1.94	7.06	1.37	Negative	26.6	1.85	1.28
	Clozapine	CLZ	5786-21-0	326.82	3.35	7.35	2.97	Negative	41.4	2.57	2.19

*K*_ow_: octanol–water partition coefficient; p*K*a: acidity constant; *D*_ow_: pH-dependent octanol–water distribution ratio; *K*_lip-w_: liposome–water partition coefficient; *D*_lip-w_: liposome–water distribution ratio at pH 7.5. Calculation processes of these parameters for eight targeted PDs described in SM Text S3.

**Table 2:** The exposure concentration, toxicokinetic parameters and BCFs of the selected chemicals in *D. magna* at pH 7.5.

Chemicals	LC_50_ (mg/L)	Exposure Concentration (mg/L)	*K*_u_ (L/kg/h)	*K*_d_ (h^−1^)	BCF_k_ (L/kg ww)	BCF_ss_ (L/kg ww)	log BCF	R^2^
CBZ	149	1.5	3.7 ± 1.01	0.15 ± 0.01	24.67	36.16	1.70	0.95
CIT	13.72	0.15	8.52 ± 1.97	0.045 ± 0.023	189.33	163.19	2.28	0.92
SER	0.98	0.001	48.00 ± 3.1	0.049 ± 0.007	979.59	1183.87	3.18	0.90
VLF	100.3	0.1	1.53 ± 0.001	0.25 ± 0.008	6.12	9.45	0.99	0.88
AMT	6.2	0.06	12.88 ± 9.89	0.040 ± 0.012	286.22	315.32	2.47	0.67
CPM	0.73	0.001	180.0 ± 6.23	0.166 ± 0.025	1084.34	1576.27	3.75	0.90
QTP	1.80	0.02	3.49 ± 0.87	0.148 ± 0.024	23.58	16.23	1.37	0.92
CLZ	2.91	0.03	6.85 ± 1.03	0.01 ± 0.007	685.00	993.76	2.06	0.90

## Data Availability

The original contributions presented in this study are included in the article. Further inquiries can be directed to the corresponding authors.

## Supplementary Materials

The following supporting information can be downloaded at: https://www.mdpi.com/article/10.3390/toxics13060481/s1, Text S1. Extraction and instrumental analysis. Text S2. Quality assurance and quality control. Text S3. Calculation procedures, details and formulas for physical and chemical properties of eight targeted PDs. Table S1 Mass spectrometry optimization parameters of target compounds. Table S2. Average recoveries, LODs and LOQs for targeted PDs. Figure S1. Relationship between log Kow and log BCF of eight targeted PDs.
