# Supplementary material for: Comparative Toxicokinetics and Biomarker Responses of Typical Psychiatric Pharmaceuticals in Daphnia magna
Source: Toxics. 2025 Jun 6;13(6):481. doi: 10.3390/toxics13060481 (PMC12197320; doi:10.3390/toxics13060481)
Supplement: Supplementary file 1 [file toxics-13-00481-s001.zip › toxics-3634658-supplementary.pdf]

Comparative toxicokinetics and biomarker responses of typical psychiatric pharmaceuticals in *Daphnia magna*

Haohan Yang[a] *, Hao Xing[a], Zhuoyu Chen[a], Linghui Kong[a], Hanyu Jiang[a]

[a] College of Environmental Science and Engineering, Yangzhou University, Yangzhou 225127, China

*Correspondence to: Haohan Yang
E-mail address: hhy@yzu.edu.cn

Supporting Information includes the following:

Number of Pages: 5

List of Texts

Text S1. Extraction and Instrumental Analysis

Text S2. Quality assurance and quality control

Text S3. Calculation procedures, details and formulas for the physical and chemical properties of eight targeted PDs

List of Tables

Table S1 Mass spectrometry optimization parameters of target compounds.

Table S2. Average recoveries, LODs and LOQs for targeted PDs

List of Figures

Fig. S1 Relationship between log K_{ow} and log BCF of eight targeted PDs.

Text S1. Extraction and Instrumental Analysis

For extraction, the collected organisms were homogenized using Pellet Pestle motor (Kontes, Vineland, NJ, USA). Then, 500 μL of methanol containing 0.2% formic acid and 10 μL of 100 mM EDTA-Na_2 were added and ultrasonic for 3 min. The samples were then filtered using a 0.22 μm nylon syringe filter (Advantec MFS, Dublin, CA, USA) for analysis. In addition, the exposure solutions were also sampled and filtered with 0.22 μm nylon syringe filters before analysis.

The targeted PDs were analyzed by a Waters Acquity UPLC-MS/MS system (Waters, Milford, MA, USA) with an electrospray ionization source (ESI) in positive ionization mode. The Waters ACQUITY UPLC–MS/MS system consist of a solvent delivery device, an autosampler, a column thermostat and the tandem quadrupole mass spectrometer XEVO TQ MS (Waters, Milford, MA, USA). An ACQUITY UPLC BEH-C18 column (100mm×1.7μm×2.1mm; Waters, USA) was employed and maintained at 40 °C. In positive ionization mode, the mobile phase consisted of eluent A (ultrapure water/ methanol + 0.1% formic acid, v/v=98/2) and eluent B (acetonitrile). The gradient program was: 90% A held for 0.2 min, decreased linearly to 5% A in 3 min and kept for 1 min, and then increased to 90% A in 1 min. An injection volume of 5 μL was used for each analysis, and the flow rate was 0.3 mL/min. Multiple responses monitoring (MRM) mode was used to identify and quantify compounds. The ESI mode, precursor ion, the product ion, the accelerator voltage, and the collision energy of each compound were listed in Table S1.

Text S2. Quality assurance and quality control

The limit of detection (LOD) and the limit of quantitation (LOQ) were defined as the 3- and 10-fold standard deviation (SD) of the average procedural blanks (n = 6), respectively. Qualified linearity for the standard calibration curves (1-500 ng/mL) was achieved with the coefficients of determination (R^2) higher than 0.99. Procedural blanks, solvent blanks and check standards (standard solution with each target compound at 100 μg/L) were run for each group of 20 samples. The relative standard deviation (RSD) values were less than 20%.

Text S3. Calculation procedures, details and formulas for the physical and chemical properties of eight targeted PDs

pH-Dependent Octanol-Water Distribution Ratio: log D_{ow} was calculated using the formula: $\log D_{ow} = \frac{\log K_{ow}}{1+10^{i(pKa-pH)}}$, where i=−1 (for anions) or i=1 (for cations, with pH = 7.5).

Ionic form using MarvinSketch 22.5, ChemAxon Ltd.

The proportion of neutral molecules (f_n) was calculated using the formula: [$f_n = \frac{1}{1+10^{i(pKa-pH)}}$, where i=−1 (for anions) or i=1 (for cations, with pH = 7.5)].

Liposome-water partition coefficient: log $K_{Lip\text{-}w}$ was queried from the website: https://www.ufz.de/index.php?en=31698&contentonly=1&m=0&lserd_data[mvc]=Public/start.

Liposome-Water Distribution Ratio: log $D_{lip\text{-}w}$ was calculated using the formula: $\log D_{lip-w} = \frac{\log K_{lip-w}}{1+10^{i(pKa-pH)}}$, where i=−1 (for anions) or i=1 (for cations, with pH = 7.5).

Octanol-Water Partition Coefficient: log K_{ow} and acidity Constant (pKa) were obtained through a literature review.

Table S1 Mass spectrometry optimization parameters of target compounds

Compounds	Precursor ions (m/z)	Sub-ion ion (m/z)	Collision voltage (V)	Collision energy (eV)	Mode	IS
CBZ	237	179	30	34	ESI+	CBZ-d_{10}
CIT	325	109	36	26	ESI+	AMT-d_5
SER	306	158	20	26	ESI+	AMT-d_3
VLF	315	44	26	10	ESI+	AMT -d_3
AMT	277	91	62	20	ESI+	AMT -d_3
CPM	318	57	30	30	ESI+	CLZ -d_4
QTP	384	220	46	36	ESI+	CLZ-d_5
CLZ	326	192	38	42	ESI+	CLZ-d_5

Table S2. Average recoveries, LODs and LOQs for targeted PDs

Compounds	r^2	Recovery rates (%)		LOQs	LODs
		10 (ng/g. ww)	100 (ng/g.ww)	(ng/g. ww)	(ng/g. ww)
CBZ	0.996	79.5	58.7	0.51	0.17
CIT	0.991	70.3	80.5	0.27	0.09
SER	0.990	140.0	72.3	0.51	0.17
VLF	0.997	65.6	78.5	0.63	0.21
AMT	0.995	153.7	77.8	0.87	0.29
CPM	0.990	72.8	89.5	0.39	0.13
QTP	0.992	166.7	65.0	0.75	0.25
CLZ	0.994	77.8	78.6	2.01	0.67

List of Figure

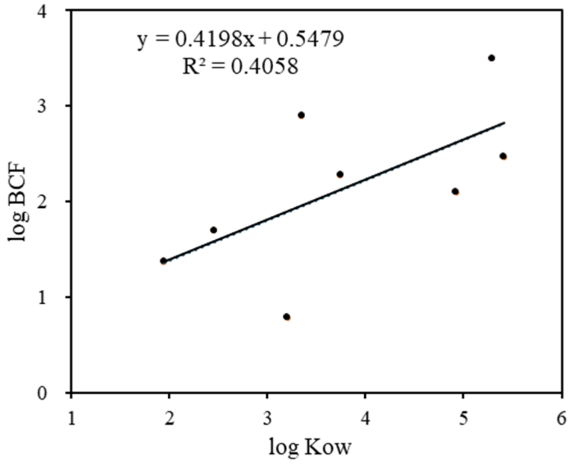

Figure S1 Relationship between log K_{ow} and log BCF of eight targeted PDs.
